# Hesitant steps from the artificial skin to organ regeneration

**DOI:** 10.1093/rb/rby012

**Published:** 2018-06-26

**Authors:** Ioannis V Yannas

**Affiliations:** Department of Mechanical Engineering, Massachusetts Institute of Technology, Cambridge, MA, USA

**Keywords:** skin regeneration, peripheral nerve regeneration, contraction blocking, dermis regeneration template

## Abstract

This is a historical account of the steps, both serendipitous and rational, that led my group of students and colleagues at MIT and Harvard Medical School to discover induced organ regeneration. Our research led to methods for growing back in adult mammals three heavily injured organs, skin, peripheral nerves and the conjunctiva. We conclude that regeneration in adults is induced by a modification of normal wound healing.

## My memorable encounter

My question to the famous trauma surgeon was simple: ‘Which is the most important problem in your field?’ Dr John F Burke, an authority on treatment of massive burns in children, replied with no hesitation: ‘A cover that can protect large wounds from infection and dehydration. A cover that will make the wound heal faster.’

In 1969, when this conversation took place in Dr Burke’s office at Shriners Burns Institute, I was 3 years away from a PhD in polymer physical chemistry earned at Princeton. After a tour of the clinic guided by Dr Burke I was ready to work on what looked to me like an urgent, and emotionally distressing to observe, but solvable physicochemical problem. At that time in my life, I knew how to synthesize polymers and measure the properties of natural polymers but I had never worked on a medical problem. My confidence was based largely on my ignorance of skin wounds and the guess that a polymeric band aid of some kind would work. I had also been told by medical authorities that no one had solved the problem that Dr Burke had described.

The solution was to be pursued simultaneously in two laboratories. It would be an empirical solution. Supported by my NIH grant, my students and I at my MIT lab would first synthesize a large number of experimental, flexible polymeric membranes. At the same time, Dr Burke’s animal lab at the hospital would be set up to score these formulations for their ability to act as covers for skin wounds in guinea pigs. With some luck, some of these polymeric membranes would make skin wounds ‘heal faster’, as Dr Burke had prescribed. At that time, almost all I knew about wound healing came from watching my own deep skin wound above the knee close slowly and painfully several days after a soccer injury when I was 9 in Athens, Greece. I had noticed one thing: my knee stopped hurting only after it had closed completely. That is almost all I knew about wound healing when I started working with Dr Burke. The detailed biochemical events of wound healing were entirely outside my experience.

You will read below a brief narrative of an unexpected discovery for treatment of burned patients first made in 1974, which became known as ‘artificial skin’, only to be recognized in the early 1980s as an instance of induced regeneration of skin in adult animals and in the human.

## A failed experiment

We started busily synthesizing several polymers in my lab and shaped them like membranes in preparation for grafting them on deep skin wounds in guinea pigs aided by Gaby Pollinger in Dr Burke’s lab. That part went slowly but smoothly. Many of the polymers, synthesized mostly by Dr Phil Gordon in my lab, could be fabricated in thickness levels that could control moisture loss as well as protect against bacteria entry into the wound. What I sorely lacked was some kind of metric that could be used to compare the efficacy in speeding up wound healing of these polymeric formulations. After agreeing to work on the project with Dr Burke, I read several articles on wound-healing processes, mostly focused on detailed biochemical studies that investigators had conducted over many years. There was no mention of speeding up healing but a lot seemed to be happening inside a wound that could be considered relevant: blood clotting, inflammatory processes, replacement of injured tissue and so on. Which of these processes would we need to modify in order to accelerate wound healing? Should we look for a particular clotting factor or active protein molecule? Or put aside these detailed processes and use a novel approach? I was now spending many hours looking at guinea pigs healing their skin wounds, often photographing them, trying to figure out how to measure how fast they were healing.

At one point I realized that the simplest feature of wound healing that I could measure with my primitive experimental means was the rate of loss in wound area by the normal process of wound contraction. Wound closure was one feature of wound healing that basic medical investigators, mostly biochemists, had largely neglected to study so far in detail—yet it seemed to be in line with the main goal in Dr Burke’s treatment of burn patients. As a result, our research efforts eventually crystallized into an empirical protocol of grafting these polymeric membranes on standardized animal skin wounds and simply measuring the kinetics of wound contraction. A large number of synthetic polymeric membranes, each from a different chemical family, were grafted on animal skin wounds and tested in this manner. We were disappointed to observe that, one after the other, these synthetic membranes had no effect whatsoever on the kinetics of wound closure. The wounds closed at normal rates with a typical half-life of contraction of about 11 days but it was as if these animals were totally unaware of their special treatments. Clearly, our synthetic polymeric membranes somehow did not interact with the wounds at all.

Having being frustrated by working with synthetic polymers, and running out of options, I decided to try collagen, a natural polymer. My familiarity with collagen and its amorphous counterpart, gelatin, traced back to the days of my doctoral thesis, when I had studied the physical chemistry of these two polymers in the solid state. In the MIT lab my doctoral student, Chor Huang, had been preparing and characterizing for his thesis research graft copolymers of collagen and certain polysaccharides, called glycosaminoglycans (GAGs). I kept referring to these copolymers as ‘biological fiber reinforced composites’ because in most of our connective tissues, collagen fibers are embedded in a matrix of GAG polymers. I was actually looking forward to studying them in detail as mechanical analogs of the extracellular matrix in our tissues. But when I faced the prospect of another ‘null’ experiment, where no effect had been registered, I started thinking that these copolymers could be next in line, possibly even the last in line.

The result of the experiment with the collagen–GAG membrane was totally baffling: Animal wounds covered with this membrane strongly slowed down their contraction, apparently slowing down healing rather than speeding it up! We were now measuring a half-life of contraction of about 30 days, much higher than the average of 11 days measured with the various synthetic polymers or with the ungrafted wounds. All of us, Dr Burke and his animal technician, my students and I were totally disillusioned but also apprehensive. We had used most of the funds in my NIH grant only to end up with what we were now labelling as a ‘failed’ experiment, a test that yielded an outcome going exactly along the wrong direction. Instead of speeding up wound closure, we had managed to slow it down.

In an extreme effort to pull something positive out of what looked like a disastrously unsuccessful experimental series, I had a desperate thought. It was the weak hope that a detailed study of the tissues in the failed experiment would somehow show what went wrong and possibly provide a clue about how to do it right the next time. After all, the collagen–GAG membranes had managed to interact with the wounds while the synthetic polymers had not. Over the next few nights, I spent many hours at the pathology lab of the hospital, looking at tissue slides of wound tissues from the ‘failed’ experiment that resulted from use of the collagen–GAG membrane. I also studied many tissue slides from the previous experiments where we had grafted synthetic polymers with no effect on the contraction rate.

Looking at tens of pathology slides one after another with largely ignorant eyes, I nevertheless noticed something strange. In all previous ‘null’ experiments that had no effect on the contraction rate, the slides of healed wounds always showed some scar tissue. In the middle of the night the hospital pathologist sitting next to me looking at his own microscope was doing his routine work for the hospital. He kept treating me like a first-year medical student. He looked at my slides, confirmed that the tissue I had been looking at was in fact scar and went back to his bench doing his own work. Yet, in slides obtained with the ‘failed’ experiment using the collagen membrane, I could not find any scar tissue. What did that mean? After a brief consultation with the pathologist I learned that wounds always make scar when they heal: they make large or small scars but scarless healing just does not happen. Slowly, the likelihood surfaced in my mind that slides from the ‘failed’ experiment included a new kind of tissue that had formed inside the wound. When I showed the slides to my hospital colleagues they named the new tissue ‘neodermis’, that is a newly formed physiological tissue similar to the dermis of normal skin, the tissue lying underneath the epidermis. In normal skin, the dermis is a tough tissue layer that protects our body from many types of mechanical damage.

At first, there was little excitement around the lab about a possible synthesis of the dermis in mammals. The prevailing opinion in the 1980s, even 1990s, strongly favored the epidermis (the outside tissue in skin), not the dermis, as the key tissue that is required in any clinical procedure for treatment of skin loss. My biological colleagues at MIT and elsewhere had been spending their research funds inventing processes that yielded epidermal grafts, to be tried as covers for skin wounds. One probable reason for this overwhelming preference for the epidermis and neglect for the dermis among life scientists at that time is a belief that persists to this day: the dermis is often viewed as an inactive matrix, a biologically inert tissue mass that lacks the quintessentially biological features that richly cellular tissues, for example epithelial tissues such as the epidermis, possess. However, it later became clear to me, mostly from published independent studies by surgeons and dermatologists, that, following injury, the lost epidermis can regenerate spontaneously (e.g. following a blistering injury following mild sunburn) while the lost dermis, for example after a deep burn, never does. This is why I concluded many years later, against prevailing opinion, that the dermis, not the epidermis, is the really valuable prize to be reaped in studies of induced skin regeneration.

Dr Burke was pleasantly surprised by this finding of scarless healing and neodermis formation and soon decided to conduct a study with 10 children who had suffered large burns and were at serious risk of dying soon. All grafts required for the clinical study were urgently prepared in my MIT lab under the round-the-clock supervision of my associates Eugene Skrabut and Dr Martha Umbreit, assisted by several students involved in various research projects. Dr Burke’s clinical study was a great success. Children’s lives were saved.

## The empirical fact of regeneration

One of our early problems was verification that the neodermis was in fact part of normal skin rather than some unusual form of scar. Neither hair follicles nor sweat glands are found in scar. Guinea pig wounds, or human wounds, that had been treated with the collagen–GAG scaffold generally looked strange, lacking hair follicles and sweat glands. So, which was it: dermis or scar? I had to find a way to ‘analyze’ this new tissue. I remembered my undergraduate days in the analytical chemistry lab at Harvard where a sample of an ‘unknown’ was distributed by the teaching assistant and the students were expected to identify it by executing a number of well-defined screening tests involving colorful precipitates. But that was not to be the case with the ‘unknown’ tissue we had synthesized. I asked a number of medical professionals in the Boston area: ‘How do you identify physiological skin?’ The survey yielded an interesting collection of approaches toward an analysis of the strange looking tissue patch on the back of the experimental animals. A surgeon told me that it should be skin if, after excision of a small area, the wound bled and then closed with formation of scar. A dermatologist thought that measurement of the moisture permeability rate through the new organ would show whether the rate of water loss was physiological or not. Other opinions from medical specialists were also heard. Many years later, Dr George Murphy, a dermatopathologist at Brigham and Women’s Hospital, found convincing ultrastructural evidence that the new tissue was in fact dermis, not scar. In an independent study, Dr Carolyn Compton, a plastic surgeon at Massachusetts General Hospital, later completed a histological study of the new tissue by focusing at the interface between the outer layer of skin (epidermis) and the inner layer (dermis): The interface is normally flat in scar but wrinkled into a wave-like pattern in the normal (scarless) dermoepidermal junction. The tissue she had synthesized in animals in her laboratory using our collagen–GAG membrane was in fact dermis, not scar.

I first became convinced that the unknown tissue was dermis rather than scar when my new pathologist colleague, Dr Robert Trelstad, suggested that we look at the controversial tissue slices with the ‘neodermis’ between cross-polarized lenses in the optical microscope. These views showed clearly the brightly birefringent collagen fibers arranged in a roughly random disarray in which they appear in the dermis, clearly distinct from the weakly birefringent and highly oriented fibers in scar tissue. Much later, Ariel Ferdman, a doctoral student in my lab who had a background in physics, developed a measurement of collagen fiber orientation based on scattering of a laser beam from thin tissue slices. These scattering patterns provided a clear measure of alignment of collagen fibers in dermis and scar, providing a secure way to distinguish quantitatively between the two tissues.

What about the epidermis? Closure of a skin wound is incomplete when only the dermis is synthesized without an epidermis above it. My doctoral student, Dennis Orgill, developed a collagen membrane seeded by centrifugation with autologous epidermal cells that led to simultaneous synthesis both of a dermis and an epidermis, almost the complete skin organ but still lacking hair follicles and sweat glands. (This was probably the first “tissue engineering” experiment performed.) The grafting method developed by Dr Burke to cover the neodermis, still used widely today in the clinic, is covering the neodermis with a very thin epidermal graft harvested by surgical excision from the patient, a method that leaves little or no scar on the site of the harvested skin. The problem with lack of hair follicles and sweat glands was solved later, when Steve Boyce and coworkers at the University of Cincinnati showed that a modification of our cell-seeding protocol for graft preparation leads to regeneration of hair follicles and sweat glands.

**Figure 1.**
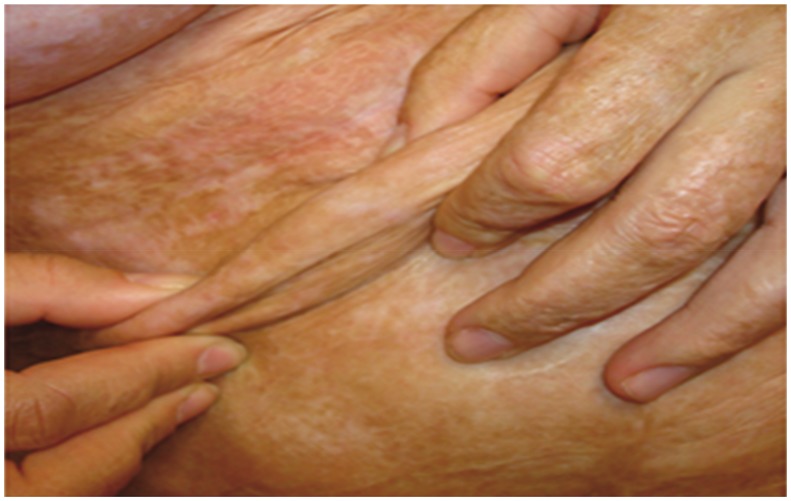
An example of the clinical use of the collagen scaffold that induces regeneration of the dermis

Since the early days of our work with Dr Burke, hundreds of thousands of patients suffering from massive skin loss have been treated with our collagen membrane in hospitals around the world. Over 340 clinical cases of skin regenerative treatments based on use of the commercial version of the collagen-GAG membrane have been published and are summarized in the attached link (http://www.ncbi.nlm.nih.gov/pubmed/?term+Integra+substitute++skin) (Fig. 1).

Success with treatment of skin wounds led us to consider working with other organs. Dr Nick Zervas, a neurosurgeon of Massachusetts General Hospital, suggested that I pursue a research direction toward treatment of brain hemorrhage. At that time, it was widely believed that the injured central nervous system (brain, spinal cord) did not regenerate at all. On the other hand, it was known that the stumps of a cut peripheral nerve (PN) (nerves in arms, legs and face) could be coaxed to reconnect by regeneration across very short experimental gaps. This approach with injured PN could only work with gaps so short that the reconnection procedure could not be generally used to treat paralyzed limbs following a severe injury. I decided to start work with the apparently less complex peripheral nervous system and asked Dennis Orgill, who had just received his doctoral degree at MIT and was now working for his MD degree at Harvard Medical School, to work on an animal model of PN injury. Our work with PN was based on the model of Goran Lundborg and coworkers, who had shown that the transected rat sciatic nerve, tubulated with silicone elastomer and with stumps separated by a gap of fixed length, could become the basis for an anatomically well-defined PN wound, suitable for quantitative experimental study. Such a model would be useful in exploring the relative ability of candidate biomaterials, in the form of experimental tubes, to regenerate nerves. Orgill, and later Lila Chamberlain, a doctoral student in my lab, showed that roughly the same collagen-based scaffold that was used to regenerate skin (minus the GAG, which turned out not to be required in the PN study) could also be counted upon to regenerate severely injured peripheral nerves across gaps of unprecedented length in rats.

Working with peripheral nerves rather than skin was a revelation to us. We found that assays for PN regeneration, both structural and functional assays, were much more highly developed and widely used than those for skin. This was partly due to the morphology of myelinated axons, which can be counted and readily lend themselves to quantitative assays of morphological outcome. In addition, electrophysiological assays of functional outcome also provided valuable quantitative information about the outcome of our experiments. Sharper assays led to sharper conclusions. For example, the superior quality of morphological data in nerve studies allowed us to develop a new metric of regeneration efficacy, the critical axon elongation. This new metric sorted out results from all sorts of experimental tubular devices that had been published by independent investigators. Using this tool, we were eventually led to a quantitative ranking of a large number of experimental tubes. We used this ranking to test theories of nerve regeneration. The increased ability to evaluate the quantitative outcome of our studies showed that the nerves that had been regenerated across long gaps in animals using tubes fabricated from porous collagen were increasingly close to being physiological, though not perfectly so.

A third organ, the rabbit conjunctiva, was also induced to regenerate in a collaborative project with Dr Peter Rubin, an ophthalmic surgeon and my doctoral student, Mark Spilker. Amazingly, the same collagen scaffold (with some modification) that worked with skin and PN also worked with the conjunctiva.

We had confirmed the *de novo* synthesis of a new dermis, new peripheral nerves and a new conjunctiva in wounds of adult mammals. Now, we had to explain just how these three organs had been regenerated.

## Chemical or biological model?

An African American reporter, visiting my MIT lab on or about 1977, was interested in writing a story about our early results with Dr Burke’s burned patients that had reached the local newspapers. Not having any deeper understanding of the phenomena we had been observing, I referred to our work at that time as ‘artificial skin’. The reporter asked me to show him our collagen membrane. I found a left-over sample that I had taken out of a desiccator sitting on the laboratory bench, prepared using an early, somewhat primitive process. It was a circular specimen of a dried up collagen membrane, diameter about 20 cm, which had lost its porosity while sitting inside the desiccator and had become opaque. It looked as if it had been cut out carefully from a paper towel, like those we use in the kitchen. The sample was starkly white. He took a photo of it.

Then the reporter pointed at the dark skin on his own hand and asked: “OK, professor, that is interesting. Do you have anything in your lab for people with my kind of skin?” Clearly, he thought, as many of us did those years, that the membrane somehow directly replaced lost skin when grafted on a deep wound, acting almost like a sophisticated band aid. Such a white band aid would look glaringly out of place on the reporter’s dark skin. I was stunned; I did not know how to answer the question. I had never seriously considered the basic biology behind our discovery. This puzzle tortured me for days. I already knew from looking under the microscope at very thin tissue slides of healed animal skin from the ‘failed’ experiment that the collagen membrane had served its purpose in a sort of self-sacrificing manner: Following grafting on animals, the membrane had slowly disappeared from tissue slices observed under the microscope, broken down to small molecules by the enzymes of the wound which typically degrade collagen fibers during wound healing. All I knew at that time was that the initially white membrane had been degraded and replaced by new living tissue, which we were trying to identify.

A week of churning the journalist’s question over in my mind passed before I stumbled toward a possible explanation: An adult mammal normally grows its own skin just once during the process of fetal development but, if badly injured later on in adult life, does not ever grow it again. Instead, adult mammals grow back scar. Was it remotely possible that our membrane had enabled the adult mammals in our experiment to grow skin for the *second* time during their lifetime? If this was true, the African American journalist would have been hypothetically able to synthesize black skin, while my white brother would synthesize white skin! This sounded like a wild idea. Yet, it now appears, over 35 years later, based on testimony from surgeons, that this guess may have been correct. If so, our ‘disastrous’ experiment in 1976 may have opened up the possibility, a slim one I thought at that time, that patients can be induced to grow back their own organs that have been damaged badly. But this was just a passing thought, with no evidence to back it up at that time, simply crafted to answer a reporter’s pointed question.

By this time I knew that I had inherited a vexing puzzle of major proportions. What was going on? Why were animal wounds grafted with the collagen scaffold healing in such an unusual way? As a chemist I had to view the neodermis as having been synthesized by cells and I cautiously referred to it in an early publication as a ‘functional extension of skin’. In the late 1970s we also replaced the term ‘membrane’ with the terms ‘scaffold’ and ‘template’ to describe what we hypothesized was a construct that hypothetically rendered steric guidance to the synthesis of the collagen fibers of the neodermis.

I was also aware of articles by biologists who described regeneration of amputated limbs in various small amphibians, such as the tadpole, axolotl and newt. Yet in my lab, we were working with experimental guinea pigs that are adult mammals, not amphibians; furthermore, we were working with skin wounds, not with amputated limbs! The adult mammal had never before been shown to regenerate any of its seriously injured organs, including the skin. Still, my conservative chemist’s approach (a noncontroversial synthesis of a tissue by cells) kept dovetailing in my mind with a biologist’s viewpoint of the phenomenon exhibited as regeneration following severe injury. Although we now recognize that these distinctions are academic to a large extent, they nevertheless inspired two experimental paradigms that were nonoverlapping and called for distinctly different experimental strategies, each using up different chunks of scarce research funds! For example, the chemist’s approach led us to search for a physicochemical characterization of the collagen scaffold, questioning or confirming the uniqueness of its molecular structure. On the other hand, the biologist’s paradigm led us to study regeneration in a developing amphibian species. Both approaches have since led to valuable mechanistic insights.

The first approach was pursued in typical physicochemical fashion. The collagen scaffold (eventually dubbed dermis regeneration template, DRT) was studied in our lab in the manner that chemists typically ‘characterize’, or identify quantitatively, any new substance that has been synthesized. Chemists typically characterize small molecules by measuring the melting point, reactivity toward standardized chemicals and so on. However, we were working with a natural polymer that required more elaborate description. The result of our study was identification of an apparently unique protein network with highly porous structure. Its uniqueness derived from the fact that its regenerative activity exhibited itself only when the average pore size (controlled by the variables of the pore-forming process) took values in the range 20–125 μm while the degradation half-life (which depends on density of crosslinking) was adjusted to about 2 weeks. Measured by Eugene Skrabut, Elaine Lee and Dennis Orgill in my lab, the levels of these two structural properties were narrowly defined. Collagen scaffolds with properties clearly outside these limits did not induce regeneration. This was evidence of high regenerative specificity. But it was, by itself, not the answer to the mechanistic question we were pursuing.

**Figure 2.**
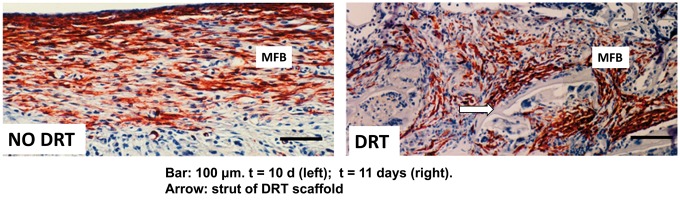
The extensive rearrangement in MFB assemblies when the scaffold is grafted on the wound

The biologist’s approach led us to study the processes by which healing of skin wounds takes place in the developing frog. We decided to forget for some tine the difference between amphibians and mammals and focused entirely on the modes of skin wound closure. We selected a developmental model because we knew in advance that wound healing took place in very different modes as the amphibian grew up from early-stage tadpole to mature tadpole and then to adult frog. In this way, we could study these different modes of wound healing while keeping the species unchanged! This study was an eye opener because it showed that closure of wounds throughout the life of this amphibian occurred by just three processes: contraction of wound edges, scar formation and regeneration. No other process for wound closure could be observed. The fractional contribution of each of these processes to wound closure could be quantified in terms of the easily measurable fraction of wound area closed by each process. The frog data, and subsequent data with other animals, including rats, led us to a couple of crucial conclusions about wound healing: first, scar formation is not an independent process; it is secondary to contraction and depends on the incidence of contraction. Both in skin wounds and PN wounds the normal mechanical field of wound contraction was found to be responsible for the high alignment of collagen fibers during scar formation. When contraction was inhibited by grafting with the contraction-blocking collagen scaffold (DRT), scar formation was abolished. In skin wounds, an approximately plane stress field is developed during normal wound contraction, which aligns the long axes of contractile fibroblasts (myofibroblasts [MFB]) along the direction of major deformation of the wound margin in the plane of the wounded skin. In turn, these aligned contractile fibroblasts synthesize collagen fibers that are aligned along the same axes as the long axes of cells that synthesized them (this basic description of collagen fiber synthesis by fibroblasts had been independently shown by DE Birk and Robert Trelstad many years before.). Scar tissue is then synthesized in the plane of the skin wound. This rationale was consistent with our finding that when the contractile field had been cancelled using DRT, the resulting tissue comprised collagen fibers that lacked orientation and showed the apparent structure of normal dermis (Fig. 2).

**Figure 3.**
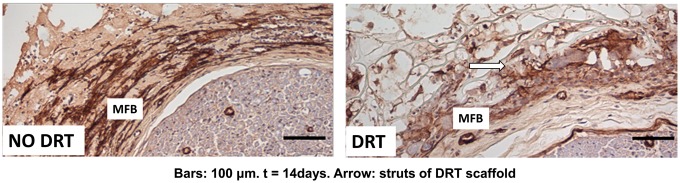
The disorganization of MFB assemblies that takes place when the transected nerve stumps are inserted into a tube fabricated from the collagen scaffold

The experience with skin wounds helped us clarify a critical phenomenon with injured peripheral nerves that had escaped notice in independent studies reported in the literature. It was known by surgeons that cutting a peripheral nerve was accompanied by shrinking of the stump diameter. These observations were not pursued further by the investigators. My doctoral student, Lila Chamberlain, in our collaboration with Myron Spector of Harvard Medical School, showed that the stumps of a cut nerve became surrounded within a few days by a thick layer (capsule) of contractile cells, which she was the first to identify as MFB. Further experimental evidence obtained by my doctoral student Eric Soller showed clearly in a quantitative study that the thickness of the capsule of contractile cells surrounding the regenerating nerve was inversely related to the diameter of the regenerated nerve and to the number of myelinated axons in it: thicker contractile capsule, smaller nerve diameter, fewer myelinated axons. It became plausible to us to hypothesize that the layer of contractile cells was applying a circumferential (compressive) mechanical field that squeezed the cross section of the nerve stump, thereby reducing the number of myelinated axons to a fraction of their physiological value, eventually leading to a poorly regenerated nerve. When the nerve stumps were inserted in a DRT tube the capsule of contractile cells practically disappeared (we found that several migrated outside though the porous tube wall), and the stumps reconnected across long gaps. We named this hypothetical sequence of events the ‘pressure cuff’ theory due to its mechanical resemblance to the common method for measuring blood pressure in a person’s arm. Comparing the process of wound contraction in these two organs, skin and peripheral nerves, we could now view them as being very similar to each other with the exception of the anatomical shape of the organ: planar in skin, cylindrical in the nerve (Fig. 3).

**Figure 4.**
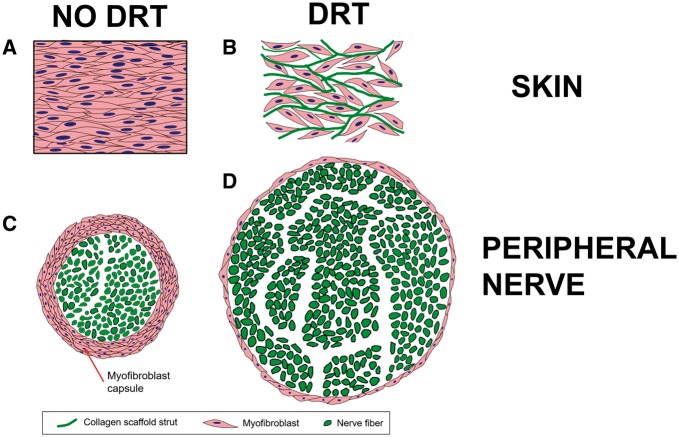
Schematically the observed changes in contractile cell phenotype taking place in skin wounds (top) and nerve wounds (bottom) when DRT is grafted. Summary: contraction blocking by DRT in skin and nerves leads to scarless healing. Mechanical field during wound contraction orients MFB long axes along major contraction axis. MFB deposit newly synthesized collagen fibrils in direction parallel to their own axes. In skin wounds, the geometry of contraction is planar; in peripheral nerve, is is cylindrical

Independent support for our theory that regeneration is induced following contraction blocking was uncovered when I searched through examples in the literature of spontaneously healing skin wounds in different animal species. These examples include the perforated rabbit ear, the injured oral mucosa in mice and swine, and skin wounds in the axolotl. In each case of these spontaneously healing wound models, the authors’ description of their experiment showed that wound contraction was either prevented due to anatomical constraints (rabbit ear) or else showed that transforming growth factor-β1, a cytokine that is required for MFB presence in wounds, was either absent or significantly downregulated. Furthermore, Eric Soller in my lab has shown that the concentration of TGFb1 in nerve wounds in the rat sciatic nerve where the stumps had been tubulated with DRT is a fraction of the value measured in the absence of DRT.

Much remains to be uncovered about this fundamental antagonistic relation between wound contraction and regeneration. Yet it is remarkable that one of our earlier, unexplained findings when using DRT was that the collagen scaffold blocked skin wound contraction. This contraction-blocking property of DRT appears to be precisely the reason why we had observed a neodermis, rather than scar, in tissue slices from the ‘failed’ experiment!

Between these two conceptual approaches, the chemical and the biological, each provided a view of their own side of the same coin. The chemical approach led to characterization of a unique polymer (protein) network that induces regeneration of skin and nerves in the adult mammal. Characterization itself did not provide an interpretation of the mechanism by which the scaffold managed to induce regeneration. It did, however, describe a well-defined structure at the molecular level that could be used to select among alternative mechanistic explanations: A given hypothesis could be screened against this information and found to be consistent with it or not. On the other side of the coin, the biological model provided a valuable clue to a rationale for these regeneration phenomena: Regeneration appeared to require blocking of wound contraction, rather than blocking of scar formation as widely thought in several fields of medicine.^1^

In summary, the original question that I had posed in the late 1970s, ‘can a person grow one of their organs for the second time’, has been answered positively for skin and peripheral nerves in adults. What is required is a modification of the wound-healing process.

## How is regeneration blocked in adult mammals?

What happens that changes so profoundly the age-old program of wound healing in adult animals? Wounds in adults have always produced scars: small scars and large scars. Scars in the skin from an injury, scars in the heart muscle from a heart attack, scars in the liver from consuming too much alcohol, scars in the kidney after repeated infection. How can this scaffold rewrite the wound-healing program all of us mammals have inherited from evolution? To answer that, we need to get a high-magnification glimpse of the normal wound-healing process, which leads to scar formation.

In untreated wounds, contractile cells, identified as MFB when they stain for alpha smooth muscle actin, comprise dense populations that form what Boris Hinz has termed specific cell–cell adherens junctions (AJ). There is evidence that macroscopic contractile events, such as wound contraction, are coordinated by AJs, via synchronization of periodic intracellular Ca^2^^+^ oscillations between MFBs that make physical contact. Furthermore, MFBs within these AJs have been shown to have their long axes aligned along a specific direction during wound contraction. A macroscopic force of about 0.1 N, measured by Higton and James, is required to close skin wounds in the rabbit. Since the force generated by an individual fibroblast is of order 1–10 nN, it seems necessary to recruit as many as 10^8^–10^9^ cells during wound contraction in a rodent in order to close a skin wound. Morphological studies by my doctoral students Karen Troxel (skin wounds) and Eric Soller (peripheral nerve wounds) have shown that, in the presence of DRT, MFBs do not form AJs nor are they dense or aligned. In nerve wounds, as in skin wounds, the presence of DRT causes the contractile cells to abandon their normal behavior (their biological phenotype) related to normal wound healing. In short, these contractile cells do not contract the wound nor do they help the wound to form scar any more. Instead of this normal scenario contractile cells synthesize new collagen fibers in a totally different pattern. The modified process yields the morphological pattern of normal dermis or of normal nerves (Fig. 3).

We are now tempted to go even deeper and ask: What is the molecular nature of the interaction between contractile cells and DRT that leads to the phenotype change? During the past few years my coworkers Dimitrios Tzeranis and Peter So at MIT have shown that the detailed chemical structure of the DRT surface plays a stunning role. In this work, contractile cells have been observed by two-photon microscopy to stick tenaciously onto specific molecular addresses, called ligands, on the surface of the collagen scaffold. The cells adhere to the ligands using integrins, the molecular extensions of cells. The specific binding that controls this adhesive interaction between cells and matrix is thought to involve the a1b1 and a2b1 integrins of the cells and the hexapeptide ligands, probably GFOGER and GLOGER, that are naturally present on the surface of collagen fibers. It is our hypothesis that these specific integrin–ligand interactions are responsible for the profound phenotype change of contractile cells that leads to blocking of wound contraction and ushers in regeneration.

Given the information presented above it is now possible to develop a plausible explanation for the structural specificity of DRT that we had found to be associated with its regenerative activity. Following injury and commencement of wound healing, contractile cells (MFB) migrate into the scaffold interior through pores that are large enough to allow them go through but small enough to provide adequate specific surface for high enough density of ligands, leading to nearly quantitative binding on the scaffold surface of the MFB present in the wound. In a competition between cell–cell binding and cell–DRT binding, the second process wins if the scaffold has the appropriate structure. Furthermore, binding between MFB integrins and surface ligands requires the simultaneous presence of the MFB and the ligand-bearing scaffold surface in the wound. This critical encounter cannot happen if the scaffold degrades too fast (before MFB have become differentiated) or too slow (e.g. after MFB have disappeared due to apoptosis). These requirements are consistent with the observed critical limits on pore size and degradation half-life of DRT (the latter was determined by my student Brendan Harley in work done for his Master’s thesis). There is, of course, also the requirement that the ligands be appropriate for the binding event, which introduces the surface chemistry of collagen as a required feature. This mechanistic explanation of DRT regenerative activity is consistent with known widespread failure to induce skin regeneration using grafts based on synthetic polymers (no surface ligands) or even collagen scaffolds with inappropriately high pore size (insufficient specific surface for binding) or very low pore size (no cell migration inside scaffold) (Fig. 4).^2^

## Open questions for future research

Over the years my laboratory at MIT has pursued the basic science that supports the novel clinical treatment that is often referred to as regenerative medicine. This work has led to a number of major questions, posed below.

A major question that is still being pursued is: Following blocking of wound contraction, precisely how is the dermis in skin wounds or the endoneurium in peripheral nerve wounds synthesized? Both of these tissues, often classified as stroma, have been highlighted above as the key objectives of an induced regenerative process since (unlike epithelial tissues) they do not regenerate spontaneously. Our preliminary observations suggest a synthetic route that credits the MFB (and, possibly, undifferentiated fibroblasts as well) with the synthesis of stroma. These cells, possibly adhering to struts of the scaffold, hypothetically synthesize new collagen fibers even as the collagen struts of the scaffold are degrading. However, a putative balance between rates of synthesis and degradation that leads to new stroma has not been adequately explored.

Even more important is the open question whether organs other than skin and peripheral nerves can be induced to regenerate in adult mammals using the rationale presented above. It would seem that organs which heal wounds by contraction and scar formation would be appropriate candidates. Even the liver, capable of regeneration of its mass (but not its shape) following surgical resection of a large fraction of its volume, closes the wound resulting from a severe injury by contraction and scar formation. Likewise, the rat kidney contracts following excisional injury and could be a candidate for regeneration using this methodology. The rat spinal cord forms scar and shows presence of contractile cells following complete transection, as shown by my doctoral student Mark Spilker, suggesting the possibility that it could be amenable to a regenerative approach. However, articular cartilage in joints lacks blood vessels and heals its wounds in a process that is distinctly different from healing of wounds in skin or nerves; it does not appear to be a candidate for regeneration by the process described here.

Organs need not be injured accidentally, as in cases of trauma, before they can be considered candidates for regeneration. An organ can be wounded electively, that is, by a deliberate surgical procedure to remove a congenitally malformed or diseased organ, followed by grafting with DRT and subsequent replacement of diseased skin by regenerated skin. Such procedures are currently used widely to treat pathological situations including the presence of congenital nevi or other cosmetic defects in skin. We emphasize here the possibility of replacing a portion of a terminally diseased organ with regenerated tissues that might suffice to function minimally yet adequately for survival and thus obviate the use of organ transplantation.

An open question concerns the age of regenerated tissues and its relation to the age of the organism itself. A recently regenerated dermis could be as old as the organism at the time of the regenerative process or it might be characterized as a younger tissue than the current age of the organism. No information is available on this issue.^3^

## Funding

Partly supported by grant RO1 NS051320 from the National Institutes of Health. 

*Conflict of interest statement*. IVY has participated in the founding of Integra LifeSciences, Plainsboro, NJ. He currently has no financial connection with the company and owns no stock of Integra LifeSciences.
